# Supplementary material for: Pathways at the Iberian crossroads: Dynamic modeling of the middle–upper paleolithic transition
Source: PLoS One. 2025 Dec 19;20(12):e0339184. doi: 10.1371/journal.pone.0339184 (PMC12716783; doi:10.1371/journal.pone.0339184)
Supplement: S1 Appendix — Includes further details for the Human Existence Potential, climate and archaeology timelines and model sensitivity tests. (PDF) [file pone.0339184.s001.pdf]

Supplementary to: Pathways at the Iberian crossroads: Dynamic modeling of the Middle–Upper Paleolithic Transition

Yaping Shao[1,*], Konstantin Klein[1], Christian Wegener[1], Gerd-Christian Weniger[2]

1 Institute for Geophysics and Meteorology, University of Cologne, Cologne & 50969, Germany
2 Institute of Prehistory, University of Cologne, Cologne & 50969, Germany

* Corresponding author. Email: yshao@uni-koeln.de

NEA and AMH Human Existence Potential

As example, Figure A shows the HEP for the NEA population under the conditions of GI11-10 and GS10-9/HE4. In GI11-10 (Fig. Aa), high-HEP regions existed in coastal areas and parts of the northern Meseta, where most MP sites are located. The areas along the north coast, the Mediterranean coast and the west coast of Portugal had the highest HEP with values exceeding 0.9. In contrast, north-western Iberia and southern Meseta had low HEP. This result is consistent with the archaeological site distribution, as no MP sites are found here. Both Mesetas have a rich data record with numerous surface sites of the Middle Pleistocene. Dated sites from the late Middle Paleolithic are rare and the absence could therefore possibly be a research gap. But long-term surveys in the western part of northern Meseta (e.g., in the Duero Basin) confirm that the absence of sites there is not due to research bias but might indicate abandonment of the area after MIS5 [1]. Although a high-HEP region, the northern Meseta shows human settlement only in its eastern part, while the western part was uninhabited, likely due to the strong alternation in HEP values between the interstadial and stadial times. A comparison of Fig. Aa and Ab reveals that while the western part of northern Mesta is a high-HEP region in interstadial times, it is a low-HEP region in stadial times. Against this backdrop, the regions of north-western Iberia, southern Meseta and the western part of northern Meseta might have acted as a climate barrier which separated the high-HEP region of the west coast of Portugal from the rest of high-HEP regions on the peninsula, making the exchanges between the NEA groups during MIS3 difficult.

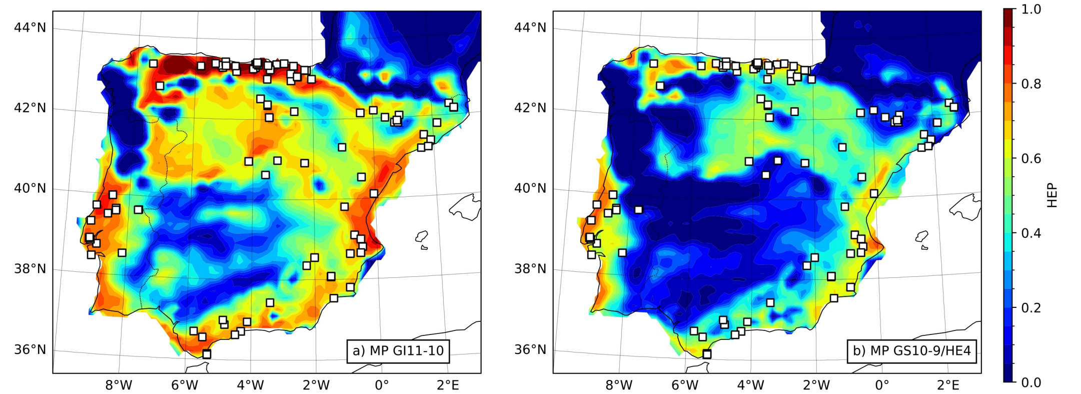

Fig A. (a) NEA Human Existence Potential for GI11-10 warm climate, and (b) for GS10-9/HE4 cold climate. White squares mark the MP sites.

During the GS10-9/HE4, the HEP for the NEA population decreased by 0.1 - 0.5 across almost entire Iberia (Fig. Ab). The decrease was more pronounced in the interior than in coastal areas,

indicating that the probability of the NEA existence in the interior of Iberia, which was already small in the GI11-10, further diminished. Several hot spots on the coastal strip retained high HEP values, which could serve as refuges for the NEAs, including the Cantabrian coast, Mediterranean coast centered around the Valencia, Gibraltar area, coast of Portugal, and small areas in the Ebro depression and adjacent northern Meseta. The HEP values in the Portuguese coastal area hardly changed, but the area suitable for the NEA existence was reduced and now surrounded by the low-HEP areas and maintaining networks to the east was almost certainly impossible.

Fig. Ba shows the HEP for the AMH population in GI11-10. Franco-Cantabria was a high-HEP region where also archaeological sites are densely distributed. In northern Iberia, high-HEP regions extended to the west coast of Spain, further than Arnero which is the westernmost site in our data set. Also, the coastal area in northern Portugal and the area to the Central System around Madrid were potentially suitable for settlement. While the Mediterranean France, reaching as far as the south of the Pyrenees, showed high probability of AMH existence, with HEP values ranging from 0.75 to 0.95. The only potential site south of the 40 Latitude is the outlier Lapa do Picareiro. Its assignment to the AUR-P1 is disputed [2].

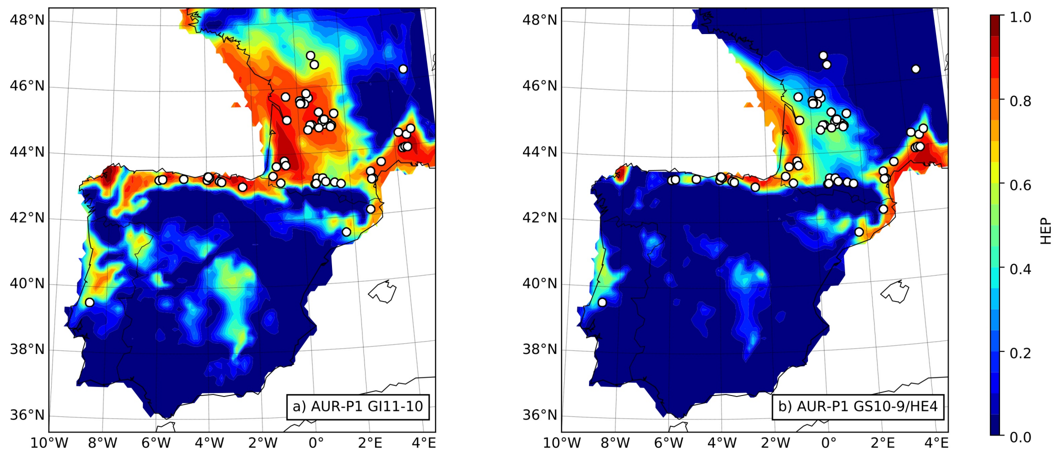

Fig B. a) HEP of the AMHs under the GI11-10 and (b) GS10-9/HE4 conditions.

In comparison to the NEAs, the AMHs lived in more humid areas with relatively low seasonal rainfall variability. In GS10-9/HE4, the HEP for the AMHs dropped sharply in large parts of the west Mediterranean region (Fig. Bb). The inland of Iberia, which was partly suited for AMH existence in GI11-10, became a hostile area in GS10-9/HE4, with the HEP values falling largely to below 0.05. The decrease occurred also in the coastal areas of Iberia. For example, the coastal areas of Portugal, favorable for AMH existence in GI11-10, almost disappeared completely. Only the Cantabrian coast and the northern most part of the Mediterranean coast retained suitable HEP.

Climate and Archaeology Timelines

Fig. C shows the climatological and archaeological timelines. The NEAs likely existed in MIS5, MIS4 and the first part of MIS3. The AUR developed in the late half of the Last Glacial Period (LGP).

Sensitivity to Model Parameters

To examine the model sensitivity to parameters, we carried out numerical tests using the configurations as summarized in Tab. A. We now discuss the results of the tests.

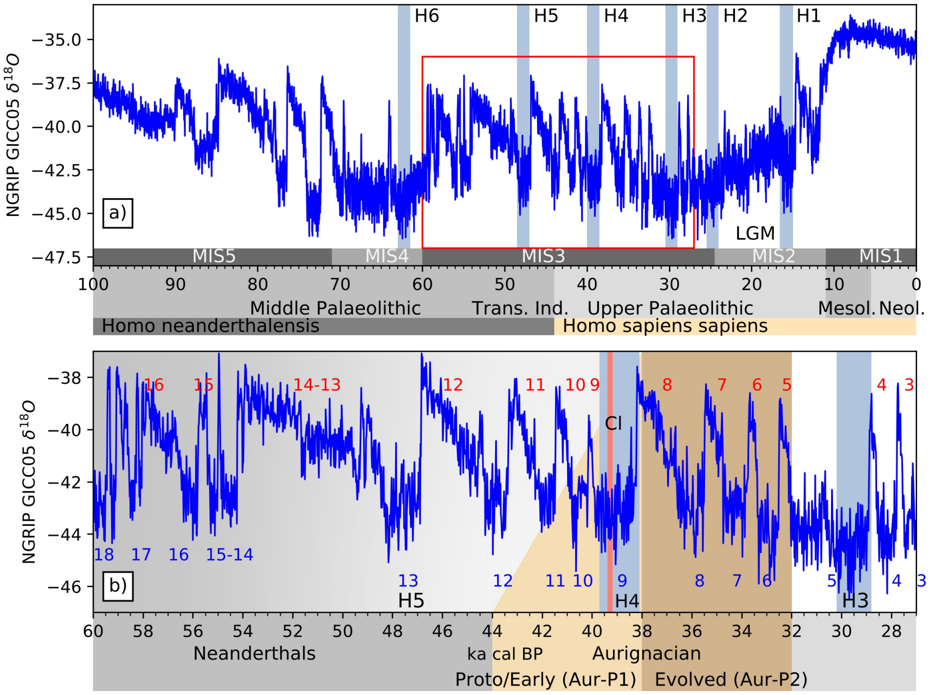

Fig C. (a) Climate during the LGP was featured by stadial and interstadial cycles, marked by Heinrich events [H5, H4 etc.] and Dangsgaard-Oeschger events. In (b), the red numbers mark the Greenland Interstadials (e.g., 10 for GI10), and the blue numbers the Greenland Stadials (e.g. 9 for GS9). Isotope δ^{18}O is a surrogate for temperature, with smaller values corresponding to warmer conditions. The abbreviations for the climate timeline include MIS for Marine Isotope Stage, LGM for Last Glacial Maximum, CI for Campanian Ignimbrite volcanic eruption. The abbreviations for the archaeological timeline, referring to the European chrono cultural divisions, include Trans. Ind. for Transitional Industries, Mesol. for Mesolithic, and Neol. for Neolithic. From [3].

Table A. The values of the parameters used for the sensitivity experiments are listed. The parameter α is a scaling acceleration; σ_u standard deviation of random motion velocity; C cultural carrying capacity; and r_B population growth parameter.

Experiment	α (km/yr)^2	σ_u (km/yr)	C (PDU)	r_B (1/yr)
Exp$_{ctl}$	1250	15	3	0.02
Exp$_r$				0.01, 0.05
Exp$_C$			1, 5	
Exp$_\alpha$	750, 1750			
Exp$_{\sigma_u}$		10, 20		

Much can be learned from the dependency of the outcomes on model parameters. As discussed in the main text, Figure 4 shows the time series of the ensemble mean NEA population size, P_{nea}, and $P_{nea} \pm \sigma_P$, with σ_P being the ensemble standard deviation.

Sensitivity to Mobility Parameter

In OW-CABM, human dispersal on microscopic scale is modeled as a diffusion process. One of the parameters for quantifying this process is the mobility parameter, σ_u, i.e., the standard deviation of human random motion. If $\sigma_u = 0$, then human dispersal is not possible, because the macroscopic drift

drives the population to a local maximum of available HEP Φ_{av}, but regions with no humans have $\Phi_{av} = 0$. Thus, the dispersal takes place as follows: humans diffuse from existing population centers to adjacent areas, making Φ_{av} there none zero, reshaping the Φ_{av} distribution and forcing a macroscopic drift in the direction of Φ_{av} gradient. The random motion is more pronounced for a larger σ_u and hence, more areas are explored.

Figure Da, b and c show the ensemble-mean population density of AMHs averaged over 42-38 ka for $\sigma_u = 10$, 15 and 20 km·yr^{-1}, respectively. The three simulations produced qualitatively similar results, with the dispersal of humans from the initially populated area in southeast France, at about (4°E, 44.08°N), to the Atlantic coast of France and Cantabria. For $\sigma_u = 10$ km·yr^{-1} (Fig. Da), the settlement covers a smaller area than for the reference case $\sigma_u = 15$ km·yr^{-1} (Fig. Db). For $\sigma_u = 20$ km·yr^{-1} (Fig Dc), the settlement covers an even larger area, with the dispersal of the AMHs reaching the west end of Cantabria and central Iberia (near Madrid), a potentially interesting hypothesis to be further tested, but supported by the neuest findings of [4]. Increased σ_u may lead to the strengthening of certain population centers (e.g., Atlantic coast of France) and weakening of others (e.g., Mediterranean coast of France).

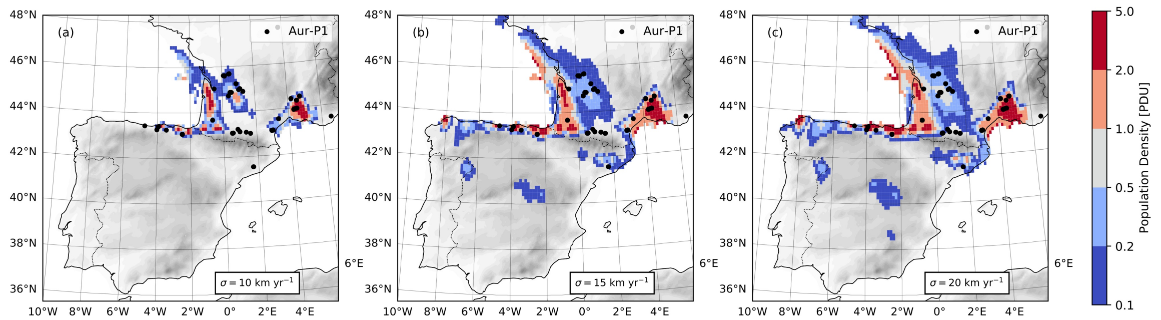

Fig D. Ensemble and time averaged population density in PDU of the AMHs in the period 42-38 ka for three different σ_u values. (a) for $\sigma_u = 10$; (b) for $\sigma_u = 15$ and (c) for $\sigma_u = 20$ km yr^{-1}.

Sensitivity to Macroscopic Drift Parameter

Figure E is as Fig D, but for $\alpha = 750$, 1250 and 1750 km^2/yr^2. A smaller α (Fig. Ea) corresponds to a wider-spread human dispersal compared to larger α values (Fig. Eb and c). While the ensemble-and-time averaged population densities show that small α appears to produce a similar result as a large σ_u, the dynamic processes the two parameters represent differ and hence also the temporal evolutions of the population patterns. Figure Ec reveals that a large α value facilitates the establishment of densely-populated centers and suppresses the dispersal caused by diffusion. As diffusion is suppressed, the population pressure is mainly balanced by the birth-death process. The choices of the two parameters (σ_u and α) balance the importance of the deterministic and random factors in the dispersal process and impacts on the population dynamics.

Sensitivity to Population Growth Rate

Figure F is as Fig D but for $r_B = 0.01$, 0.02 and 0.05 yr^{-1}. The parameter r_B strongly influences the population size, pattern and hence human dispersal. A smaller r_B leads to a smaller population size which in turn prohibits human expansion (Fig. Fa). In Fig. Fb, stable population centers emerged at the Atlantic coast of France and Cantabria, but the original population center in southwest France became weaker. This a surprising result, showing the complexity of the population dynamics and human dispersal. A detailed examination of population evolution shows that this region experienced a strong population boost at the start, causing an over population and strong human migration to the surrounding areas. The favorable HEP in the newly discovered areas continue to attract humans from

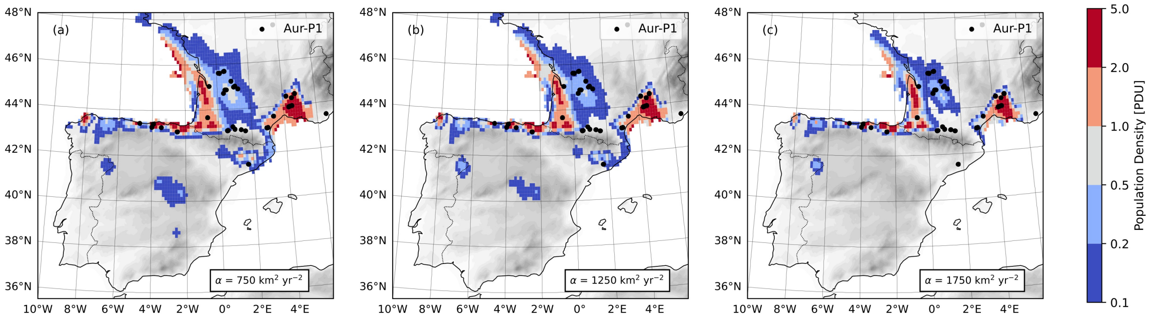

Fig E. As Fig D, but for (a) $\alpha = 750$, (b) 1250 and (c) 1750 $\text{km}^2 \cdot \text{yr}^{-2}$.

the original center. Eventually, a new high population region along the Franco-Cantabrian coast replaced the original center. In the case of Fig. Fc, an even larger population size is achieved.

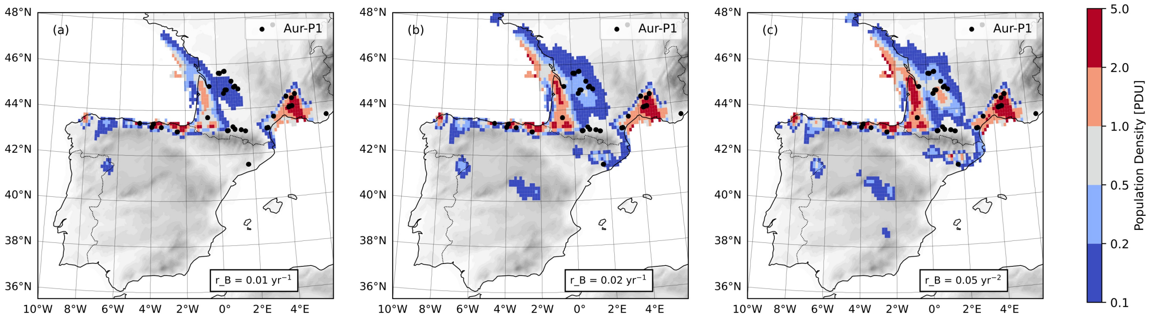

Fig F. As Fig D, but for (a) $r_B = 0.01$, (b) 0.02 and (c) 0.05 yr^{-1}.

Sensitivity to Cultural Carrying Capacity

The cultural carrying capacity C also influences strongly the population size. Figure Ga, b and c show the AMH population distribution simulated with $C = 1$, 3 and 5 PDU, respectively. The basic features for the three cases are qualitatively similar, but the populated areas, population size and settlement time fraction are all proportional to C. The increase in C enables humans to settle in region of lower HEP.

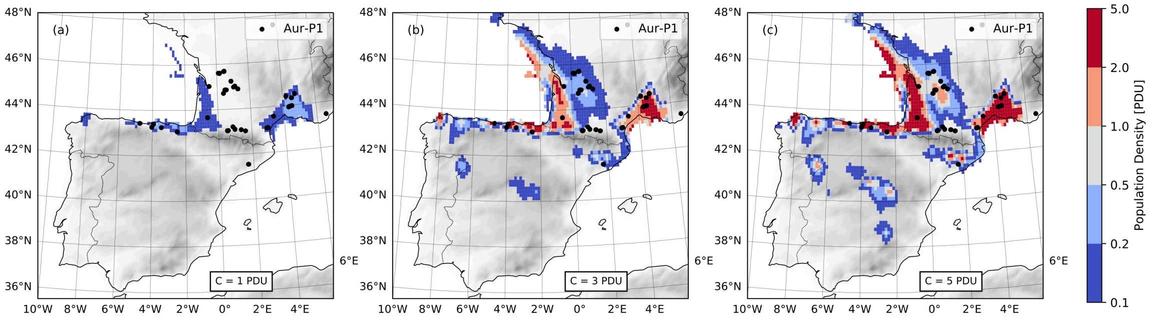

Fig G. As Fig. D, but for (a) $C = 1$, (b) 3 and (c) 5 PDU.

Sensitivity to Initial Condition

The OW-CABM simulations require an initial population distribution. For the NEA simulations presented in the main text, the initial population, N_0, was set to 1000 humans. These individuals were distributed according to a Gaussian pattern around four population centers, located at (3.93°W, 42.3°N), (0.72°W, 40.03°N), (5.54°W, 36.66°N), and (8.6°W, 39.2°N), with a standard deviation, σ_{N0} of 1°.

We tested the model sensitivity to this initial condition by varying N_0, σ_{N0}, and the locations of the population centers. Varying N_0 changes the total number of individuals, while varying σ_{N0} alters their spread; a larger σ_{N0} results in a more dispersed distribution. A total of six experiments were conducted: one control and five sensitivity tests, as detailed in Table B.

Since the Gaussian distribution places a fraction of the initial population over the ocean—where they are excluded—the effective starting population on the Iberian Peninsula is always less than the specified N_0. For example, in Exp N03000, we set σ_{N0} to 3° and increased N_0 to 3000 to compensate for the wider spread. Experiments N01000a and N01000b instead test four alternative population centers, with σ_{N0} set to 3° and 1°, respectively. All experiments use the same model parameters as ExpNEA-C (Table 2) and consist of an ensemble of 100 runs each.

Table B. Sensitivity experiments to initial NEA population distribution.

Exp Name	N_0	Center 1	Center 2	Center 3	Center 4	σ_{N0}
N01000 (Ctl)	1000	3.93°W, 42.3°N	0.72°W, 40.03°N	5.54°W, 36.66°N	8.6°W, 39.2°N	1°
N0500	500	- (as above)	-	-	-	-
N02000	2000	-	-	-	-	-
N03000	3000	-	-	-	-	3°
N01000a	1000	8.0°W, 41.0°N	2.0°W, 42.0°N	2.0°W, 39.0°N	6.0°W, 38.0°N	-
N01000b	1000	-	-	-	-	1°

Figure H shows the sensitivity of the NEA population to initial conditions, presenting the total population time series (50–38 ka) for the experiments in Table B. The results indicate that the initial conditions noticeably influence the model outcomes only in the first 500 to 1000 years. Beyond this spin-up period, all experiments yield very similar results in both ensemble mean and standard deviation. The variance among the different experiments is much smaller than the ensemble standard deviation within any individual experiment. This shows that the specification of the initial conditions has only a limited impact on the long-term model results. Consequently, for our discussion of NEA population dynamics in the main text, we set the model spin-up time to 500 years and excluded this period from the analysis.

Sensitivity of NEA Human Existence Potential to Exclusion of Chât Sites

As discussed in the data section, debates persist regarding the inclusion of Chât sites in the Mousterian (MP) sample as evidence of NEA presence. To test the sensitivity of our results to this uncertainty, we recalculated the HEP for the NEA population during GI11-10 and GS10-9/HE4 with Chât sites excluded (Fig. I). Comparison with Fig. A reveals nearly identical HEP patterns, with only minor differences in magnitude.

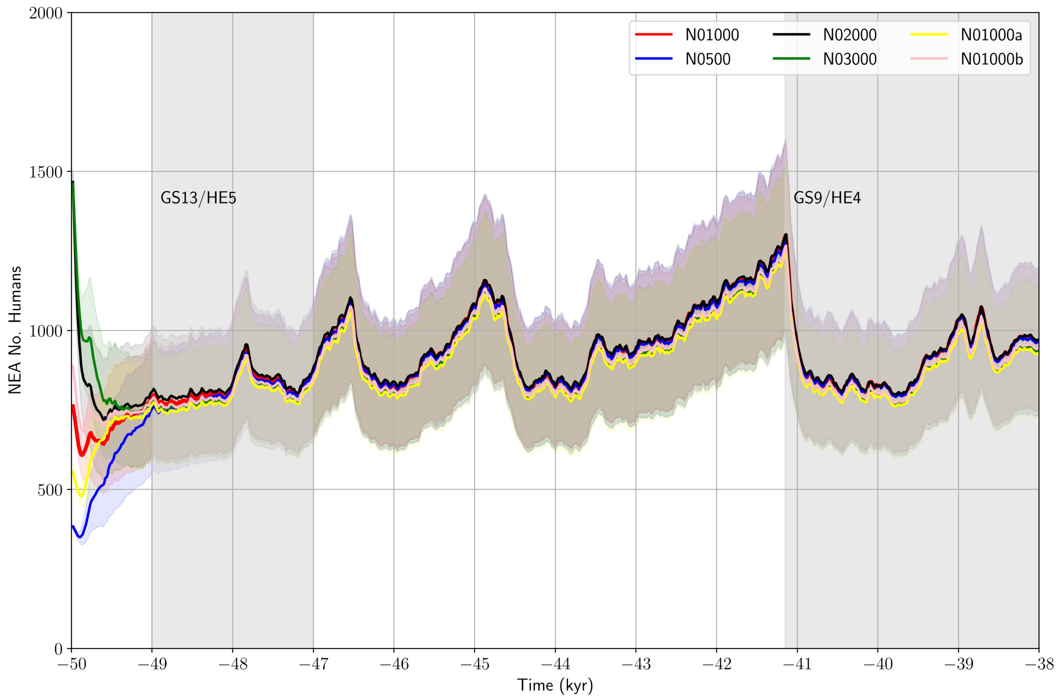

Fig H. Sensitivity of the NEA population to initial conditions. Time series of the total NEA population (50–38 ka) are shown for the ensemble experiments listed in Table B. The thick lines denote the ensemble means, and the shaded areas indicate the range of one standard deviation above and below the mean.

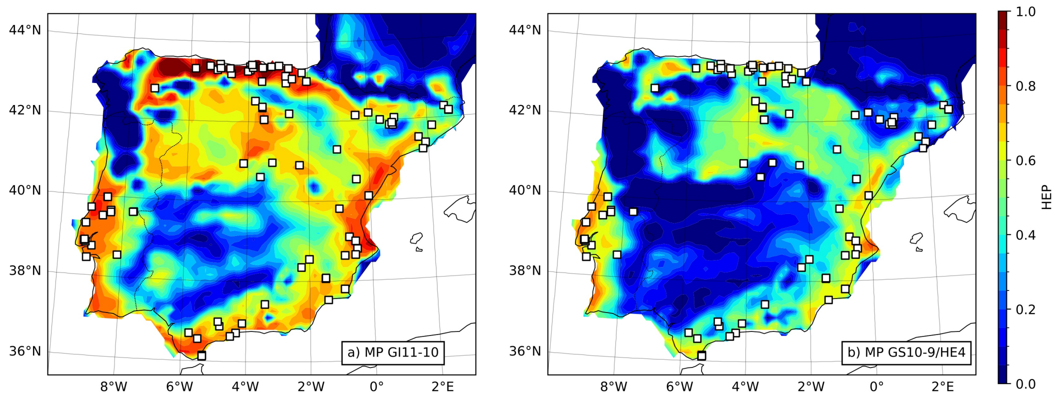

Fig I. As Fig. A, but with the Châtelperronian sites excluded from the HEP model training.

Table C. List of the MP and CHÂT sites used in this study, which is an excerpt of the MIS3 MP sites documented in [5]. The CHÂT sites are in italics

Site Name	Site Type	Site Name	Site Type
Abauntz	Cave	Millán, Cueva	Cave
Abric Romani	Abri	Mira Nascente	Open Air
AGP5	Cave	*Mirón, El*	Cave
Almonda	Cave	Molino, Abrigo del	Abri
Amalda	Cave	Mollet I	Cave
Anton, Cueva	Cave	Morín, Cueva	Cave
Arbreda, Cueva de L'	Cave	Moros de Gabasa, Cueva de los	Cave
Ardales, Cueva de	Cave	Muricecs, Cova dels	Cave
Arenillas, Covacho de	Unknown	Negra, Cova	Cave
Arrillor Cave	Cave	Niño, Cueva del	Cave
Axlor (Azlor/Axlor, Cueva de)	Cave	Oliveira, Gruta da	Cave
Bajondillo, El	Cave	Otero, El	Cave
Beneito, Cueva	Cave	Palomar, El (Albacete)	Abri
Boja, La	Abri	Palomas (del Cabezo Gordo), Sima de las	Cave
Buraca Escura	Cave	Pego do Diabo	Cave
Buraca Grande	Cave	Peña Cabra	Abri
Caldeirão, Gruta do	Cave	Peña Miel	Cave
Cantavieja, Cueva de los toros	Cave	*El Pendo*	Cave
Carigüela/Carihuela, Cueva de la	Cave	Picareiro, Lapa do	Cave
Casares, Los	Cave	Prado Vargas, Cueva de	Cave
Castillo, El	Cave	Quebrada, Abrigo de la	Abri
Cochino	Cave	Rexidora Cave	Cave
Coll Verdaguer, Cova del	Cave	Roca dels Bous	Abri
Columbeira	Cave	Ruso, El	Cave
Conceicáo	Open Air	Salemas quarry	Open Air
Conde (Forno), Cueva del	Cave	Salemas, Gruta de	Cave
Covalejos	Cave	Salt, El	Cave
Cuco, El	Abri	San Cristobal, Fuentes de	Abri
Dalt del Tossal de la Font, Cova de	Cave	Santa Linya, Cova Gran de	Cave
Devil's Tower	Cave	Santimamiñe, La cueva de	Cave
Eirós, Cova	Cave	Sidrón, El	Cave
Ekain	Cave	Sima de las Palomas de Teba	Abri
Ermita, Cueva de la	Cave	Sopeña, Abrigo de	Abri
Ermitons, Cueva de los	Cave	Teixoneres Cave	Cave
Escoural 3, Gruta do	Cave	Trucho, Fuente del	Cave
Esquilleu, Cueva del	Cave	Valdegoba	Cave
Estret de Tragó, Cova del	Cave	*Valiña, A*	Cave
Figueira Brava, Gruta da	Cave	Valle de las Orquideas	Open Air
Finca Doña Martina	Abri	Vanguard Cave	Cave
Flecha, Cueva de la	Cave	Vilas Ruivas	Open Air
Foradada (Calafell, Cova)	Cave	Viña, La	Abri
Foz do Enxarrique	Open Air	Zafarraya, Cueva del Boquete de	Cave
Fuentes, Las	Unknown		
Gegant, Cova del	Cave		
Gorham's Cave	Cave		
Güelga, La	Cave		
Higueral de Valleja Cave	Cave		
Higueral-Guardia de Motillas	Cave		
Hornos de la Peña	Cave		
Hotel California	Open Air		
Ibex Cave	Cave		
Jarama VI	Cave		
Kurtzia	Open Air		
Labeko Koba	Cave		
Lapa dos Furos	Cave		
Lezetxiki	Cave		
Llonin Cave	Cave		

Table D. List of the Aurignacan Phase 1 sites used in this study, which is an excerpt of the Aurignacian sites documented in [6].

Site Name	Site Type
Abric Romani	Cave
Aurignac II	Cave
Barbas I et III	Open Air
Caminade	Cave
Castanet, Abri	Abri
Cellier, Abri	Abri
Chaise, La	Open Air
Combe Saunière	Cave
Corbiac- Vignoble	Open Air
Covalejos	Cave
Crouzade, La	Cave
Esquicho Grapaou	Cave
Figuier, Grotte du	Cave
Fontéchevade	Cave
Garet	Open Air
Gargas	Cave
Gourdan (El), Grottes de	Cave
Graulet VI, La	Open Air
Labeko Koba	Cave
Laouza, La	Cave
Mandrin, Grotte	Cave
Mas d'Azil, Grotte du	Cave
Otero, Cueva del	Cave
Pair Non Pair	Cave
Pêcheurs	Cave
Picareiro, Lapa do	Cave
Pont Neuf, Le	Cave
Rothschild, Abri	Abri
Souquette, Abri de la	Abri
Tarté	Cave
Traouc de la Fado	Open Air
Tutto de Camayot (La Tuto de Camalhot)	Cave

References

1. Sánchez Yustos P, Diez Martín F. Dancing to the rhythms of the Pleistocene? Early Middle Paleolithic population dynamics in NW Iberia (Duero Basin and Cantabrian Region). Quaternary Science Reviews. 2015 Aug;121:75-88. Available from: https://linkinghub.elsevier.com/retrieve/pii/S0277379115002048. doi:10.1016/j.quascirev.2015.05.005.

2. Zilhão J. The late persistence of the Middle Palaeolithic and Neandertals in Iberia: A review of the evidence for and against the Ebro Frontier model. Quaternary Science Reviews. 2021;270:107098. Available from: https://www.sciencedirect.com/science/article/pii/S027737912100305X. doi:https://doi.org/10.1016/j.quascirev.2021.107098.

3. Shao Y, Limberg H, Klein K, Wegener C, Schmidt I, Weniger GC, et al. Human-existence probability of the Aurignacian techno-complex under extreme climate conditions. Quaternary Science Reviews. 2021;263:106995. Available from: https://www.sciencedirect.com/science/article/pii/S027737912100202X. doi:https://doi.org/10.1016/j.quascirev.2021.106995.

4. Sala N, Alcaraz-Castaño M, Arriolabengoa M, Martínez-Pillado V, Pantoja-Pérez A, Rodríguez-Hidalgo A, et al. Nobody's land? The oldest evidence of early Upper Paleolithic settlements in inland Iberia. Science Advances. 2024;10(26):eado3807. Available from: https://www.science.org/doi/abs/10.1126/sciadv.ado3807. arXiv:https://www.science.org/doi/pdf/10.1126/sciadv.ado3807. doi:10.1126/sciadv.ado3807.

5. Rotgänger M, Blumenröther J, Bradtmöller M, Kehl M, Otto T, Pastoors A, et al.. CRC806 C1 Database Iberia Late Middle Palaeolithic to Magdalenian [dataset]. CRC806-Database; 2021. doi:10.5880/SFB806.67.

6. Schmidt I. CRC806 E1 AUR Sites Database 20210331 [dataset]. CRC806-Database; 2021. Available from: https://doi.org/10.5880/SFB806.63. doi:10.5880/SFB806.63.
